# Statistical analysis plan for the Recovery-focused Community support to Avoid readmissions and improve Participation after Stroke randomised controlled clinical trial

**DOI:** 10.1186/s13063-023-07864-2

**Published:** 2024-01-23

**Authors:** Monique F. Kilkenny, Muideen T. Olaiya, Janette Cameron, Natasha A. Lannin, Nadine E. Andrew, Amanda G. Thrift, Maree Hackett, Ian Kneebone, Avril Drummond, Vincent Thijs, Olivia Brancatisano, Joosup Kim, Megan Reyneke, Shaun Hancock, Liam Allan, Fiona Ellery, Geoffrey Cloud, Rohan S. Grimley, Sandy Middleton, Dominique A. Cadilhac, on behalf of the Recovery-focused Community support to Avoid readmissions and improve Participation after Stroke (ReCAPS) investigators, Dominique Cadilhac, Natasha Lannin, Helen Dewey, Monique Kilkenny, Nadine Andrew, Ian Kneebone, Avril Drummond, Jan Cameron, Amanda Thrift, Maree Hackett, Christopher Levi, Mariko Carey, Geoff Cloud, Rohan S. Grimley, Sandy Middleton, Vincent Thijs, Toni Aslett, Jonathon Li, Ernest Butler, Henry Ma, Pamela Galindo, Mark Gocotano, Andrea Moore, Fides Camino, Lily Murphy, Michael Teodoro, Bronwyn Coulton, Louise Lee, Philip Choi, Claire Buchanan, Tessa Busch, Darshan Ghia, Phoebe Lee, Gillian Edmonds, Rowena Singkang, Berzenn Urbi, Nicola Hall, Marie Matanas, Rebecca Danton, Natasha Bonanno, Kylie Tastula, Erin Li, Lucy Nolan, Timothy Kleinig, Jennifer Cranefield, Rajesh Khanna, Kirsty Page, Disha Patel, Kelly Jones, Sarah Dennien, Donna Rowley, Suzanne McGufficke, Rohan S. Grimley, Muideen Olaiya, Jonathan Li, Rebecca Barnden, Amanda Elston, Tara Purvis, Graeme Hankey, Leonid Churilov, Geoff Donnan, Coralie English, Jan Cameron, Olivia Brancatisano, Dawn Harris, Megan Reyneke, Lana Coleman, Tharshanah Thayabaranathan, Sue Mosely, Shaun Hancock, Oluwatobi Afolabi, Verena Schadewaldt, Toni Withiel, Fiona Ellery, Toni Aslett, Lisa Murphy, Eleanor Horton, Brenda Booth, Ida Dempsey

**Affiliations:** 1 Stroke and Ageing Research, Department of Medicine, School of Clinical Sciences at Monash Health, Monash University, Clayton, Australia; 2 Stroke Division, Florey Institute of Neuroscience and Mental Health, University of Melbourne, Heidelberg, Australia; 3 Department of Neurosciences, Central Clinical School, Monash University, Melbourne, Australia; 4 Alfred Health, Melbourne, Australia; 5 Department of Medicine, Peninsula Clinical School, Central Clinical School, Monash University, Clayton, Australia; 6 National Centre for Healthy Ageing, Monash University, Frankston, Australia; 7 The George Institute for Global Health, Faculty of Medicine, University of New South Wales, Sydney, Australia; 8 Graduate School of Health, Faculty of Health, Graduate School of Health, University of Technology Sydney, Ultimo, Australia; 9 Faculty of Medicine and Health Sciences, University of Nottingham, Nottingham, UK; 10 Department of Medicine, Austin Health, Heidelberg, VIC Australia; 11 Department of Neuroscience, Central Clinical School, Monash University, Clayton, Australia; 12 Stroke Services, Alfred Health, Melbourne, Australia; 13 School of Medicine and Dentistry, Griffith University, Birtinya, Australia; 14 Nursing Research Institute, St Vincent’s Health Network Sydney and Australian Catholic University, Sydney, Australia

**Keywords:** Stroke, Digital health, eHealth, Statistical analysis plan, Randomised controlled clinical trial

## Abstract

**Background:**

Unplanned hospital presentations may occur post-stroke due to inadequate preparation for transitioning from hospital to home. The *Re*covery-focused *C*ommunity support to *A*void readmissions and improve *P*articipation after *S*troke (ReCAPS) trial was designed to test the effectiveness of receiving a 12-week, self-management intervention, comprising personalised goal setting with a clinician and aligned educational/motivational electronic messages. Primary outcome is as follows: self-reported unplanned hospital presentations (emergency department/admission) within 90-day post-randomisation. We present the statistical analysis plan for this trial.

**Methods/design:**

Participants are randomised 1:1 in variable block sizes, with stratification balancing by age and level of baseline disability. The sample size was 890 participants, calculated to detect a 10% absolute reduction in the proportion of participants reporting unplanned hospital presentations/admissions, with 80% power and 5% significance level (two sided). Recruitment will end in December 2023 when funding is expended, and the sample size achieved will be used. Logistic regression, adjusted for the stratification variables, will be used to determine the effectiveness of the intervention on the primary outcome. Secondary outcomes will be evaluated using appropriate regression models. The primary outcome analysis will be based on intention to treat. A *p*-value ≤ 0.05 will indicate statistical significance. An independent Data Safety and Monitoring Committee has routinely reviewed the progress and safety of the trial.

**Conclusions:**

This statistical analysis plan ensures transparency in reporting the trial outcomes. ReCAPS trial will provide novel evidence on the effectiveness of a digital health support package post-stroke.

**Trial registration:**

ClinicalTrials.gov ACTRN12618001468213. Registered on August 31, 2018.

SAP version

1.13 (October 12 2023)

Protocol version

1.12 (October 12, 2022)

SAP revisions

Nil

**Supplementary Information:**

The online version contains supplementary material available at 10.1186/s13063-023-07864-2.

## Introduction

About one in three people discharged from hospital after an acute stroke experience an emergency department presentation or an unplanned readmission within 90 days of discharge [1]. Unplanned hospital readmissions are often related to suboptimal preparation and support of survivors or their carers in transition from hospital to home [1, 2], including lack of ongoing self-management support to assist with ongoing disability or complications after stroke [3]. Therefore, there is an urgent and unmet need for innovative, accessible self-management support and education programmes that align with the recovery and lifestyle goals of people living with stroke [4].

In the *Re*covery-focused *C*ommunity support to *A*void readmissions and improve *P*articipation after *S*troke (ReCAPS) trial, adults with stroke are randomised to either receive a 12-week digital health self-management support package within 2 weeks of discharge from hospital to home or control [5]. The intervention comprises personalised goal setting with a clinician within 14 days of returning home and assignment of educational or motivational messages to support self-management and goal attainment that allow for progression in skill development. The messages are delivered via a short message service (SMS) or email depending on the preferred contact method of the participant. The control group receives up to seven administrative text messages (e.g. a link to the Stroke Foundation website), but no healthcare messages. The primary hypothesis is that, compared to control participants, there will be a 10% reduction in the proportion of intervention participants who had unplanned hospital presentation (emergency department/admission) within 90 days after randomisation. The main secondary outcomes include goal attainment, self-efficacy, self-management, education attainment, unmet needs, resources used, mood, and quality of life, at 90-day post-randomisation. We present the statistical analysis plan for the ReCAPS trial.

## Methods

This statistical analysis plan has been written according to the “Guidelines for the Content of Statistical Analysis Plans in Clinical Trials” [6]. The study protocol has been described in detail previously [5] and is briefly outlined in the sections below. In addition, details of the initial study design, and any subsequent changes made, have been published on the Australian and New Zealand Clinical Trials Registry (number: ACTRN12618001468213). This statistical analysis plan comprises details of approaches to be used for the analysis of primary and secondary outcomes.

### Trial design

This is a prospective, multicentre, randomised controlled trial, with 1:1 allocation ratio, blinded assessment of outcomes, and intention-to-treat analysis.

### Randomisation and blinding

Randomisation is undertaken through the REDCap online system [7], with stratification balancing by age (< 65 or 65+ years) and level of disability (based on a baseline modified Rankin Scale [scores 0–2 for none-minor disability, 3–4 for moderate-severe disability]). The randomisation table, comprising the allocation sequence, block sizes, and stratification balancing, was developed outside of REDCap by an independent data analyst and imported into the REDCap study database.

The trial has a double-blind design. Therefore, hospital staff, participants, outcome assessors, and trial biostatisticians are unaware of group allocation. To ensure that blinding of participants to group allocation is maintained, the trial is described in the patient information and consent form in general terms as “providing post-hospital discharge support” [8]. Specifically, intervention approaches were broadly described in the patient information and consent form as including the “setting of specific recovery goals with trained clinicians, receiving electronic self-management information sent via SMS or email, and participation in follow-up assessments”. Outcome assessors also use an interview script to standardise outcome assessments undertaken by telephone interview and are trained to avoid entering into general discussions. To ensure hospital clinicians and participants are unaware of the allocation group, all eligible consenting patients complete goal setting, using the ReCAPS “goal setting menu”, and data collection at baseline is standardised. The trial biostatistician who will undertake the analysis is also blinded to group allocation.

### Sample size calculation

At the time we designed the trial, we were required to use indirect evidence to estimate the potential effect size of our novel intervention for the primary outcome. We estimated a sample size of 890 participants (445 participants in each intervention group) to allow sufficient power for the primary outcome analysis. The power calculation was based on the following: (a) a conservative estimate (33%) of participants in the control group having unplanned hospital presentations (emergency department/admission) within 90 days, based on data from the *Australian Stroke Clinical Registry* (*AuSCR*) linked to hospital emergency department presentations and admissions data in four states [1] and adjusted for the study inclusion criteria (being aged ≥ 18 years and discharged to home), (b) a 10% absolute reduction in unplanned hospital presentation (emergency department presentations or readmissions) within 90 days of randomisation in the intervention group vs. controls [9], (c) a ≥ 80% power at the significance threshold of *α* = 0.05 two-tailed and (d) an assumed attrition rate of 20% due to drop-out, refusal or loss to follow-up. Using an adaptive sample-size procedure we had pre-planned to re-estimate the sample size once, two-thirds of the original sample size outcomes had been obtained. Issues with recruitment during the COVID-19 pandemic and the current recruitment rate and available budget mean that this sample size is now infeasible to achieve.

The trial will be closed to recruitment by the end of December 2023, when our funding is expended with whatever number of participants is obtained by that time. This number will be used without any re-estimation of power. Using the observed data on retention rate (currently 93%), and more recent data on unplanned hospital presentations/readmissions from another study [10], our sample may provide sufficient power for a 10% difference in the primary outcome.

### Framework

All outcome analyses will be conducted to determine the effectiveness of the ReCAPS intervention over the control intervention (described in detail below).

### Statistical interim analyses and stopping guidance

An independent Data Safety and Monitoring Committee (DSMC) was established to safeguard the interests of trial participants, by assessing the safety of the intervention during the trial, and the general progress of the trial. The committee comprises one neurologist, a senior academic physiotherapist who has expertise in digital health randomised clinical trials and a senior clinical trials biostatistician. Specifically, the role of the DSMC is as follows: (a) periodically monitor and review participant safety in the trial, (b) monitor effectiveness, and (c) review participant recruitment, accrual, retention, and withdrawal.

The DSMC met at least once per year until November 2020 and have met twice per year subsequently, with a total of seven meetings as of September 2023. The committee recommended the trial to continue based on the interim analysis of the primary outcome from the first 50 participants (~6% of the estimated sample size). All interim analyses are being undertaken by a data analyst who is blinded to the group allocation. The principal investigator has the responsibility to report data on any severe adverse events to the DSMC and the independent medical monitor, if needed. The medical monitor is a neurologist who reviews serious adverse events, if deemed to be related to the intervention, and adjudicates on unplanned/planned hospital readmissions. The DSMC is also tasked with formulating recommendations relating to the selection/recruitment/retention of participants, participant management, improving adherence to protocol-specified regimens, and the procedures for data management and quality control based on these interim analyses.

There are no strict stopping criteria, but the DSMC have a responsibility to provide recommendations about continuing, modifying, or stopping the trial, in line with the DSMC charter. Following an interim analysis, the trial may be stopped for safety reasons without rejecting any null hypotheses, i.e. there is no planned adjustment of the significance level due to interim analyses. 

### Timing of outcome assessments and final analysis

The primary outcome assessment is undertaken at 90 days (~13–15 weeks) after randomisation. The schedule of assessments for secondary outcomes ranges from baseline to 90 days post-randomisation and 12 months post-randomisation (Table 1). All outcome analyses will commence after all assessments and evaluations are completed.

**Table 1:** List of study outcome

Assessment	Baseline	Follow-up^a^	
		90 days	12 months
Primary outcome			
Unplanned hospital presentation^b^		✓	
Secondary outcomes			
Goal attainment post-stroke^c^		✓	
Stroke self-efficacy^c^	✓	✓	
Anxiety and depression^c^	✓	✓	
Number of hospital contacts^d^		✓	✓
Health-related quality of life^c^	✓	✓	✓
Cost-effectiveness^c,d^	✓	✓	✓
Composite outcome^d,e^		✓	✓
Health education assessment post-stroke^c^	✓	✓	
Self-management post-stroke^c^	✓	✓	
Healthcare resource utilisation^c,d^	✓	✓	✓
Modified Rankin Scale^c^	✓	✓	
Long-term unmet needs^c^	✓	✓	

^a^Undertaken by blinded ReCAPS researchers at 90 days and 12 months post-randomisation. ^b^Includes emergency department presentations and unplanned hospital admission. ^c^Option for self-assessment by participants. ^d^Obtained through self-report and linkage with administrative data. ^e^Includes recurrent stroke, cardiovascular events, or deaths

## Statistical principles

### Confidence intervals and p-values

Statistically significant results will be identified using two-sided 5% significance levels. Estimates of this trial will be reported with 95% confidence intervals.

### Adherence and protocol deviations

Intervention fidelity will be assessed throughout the trial at both the research-team and practitioner-patient level and will be monitored throughout the trial by an external research team. This will include monitoring goal setting procedures, telephone interviews, the dispatch logs from the electronic messaging gateway, and follow-up assessments. The intervention fidelity procedures have been developed to address five key areas of the study: (a) study design, (b) training documents and processes, (c) delivery of the ReCAPS intervention, (d) receipt of intervention as per protocol, and (e) adaptations that occur to any protocol processes throughout the study (Supplemental Table I). This approach is consistent with the Behaviour Change Consortium treatment fidelity recommendations [11].

Any participant treated in a manner that deviates from the protocol may be excluded from per-protocol analyses. The nature and reasons for any protocol deviation are recorded in the electronic case report form (eCRF).

### Analysis populations

Analysis of the primary outcome will be based on the principle of intention to treat and will comprise all randomised patients. Further per-protocol analyses will be undertaken among participants who complete at least 10 of 12 weeks of the intervention to which they were randomised, irrespective of the number of goals achieved or messages received.

## Trial population

### Screening, eligibility, and recruitment

**Fig. 1:**
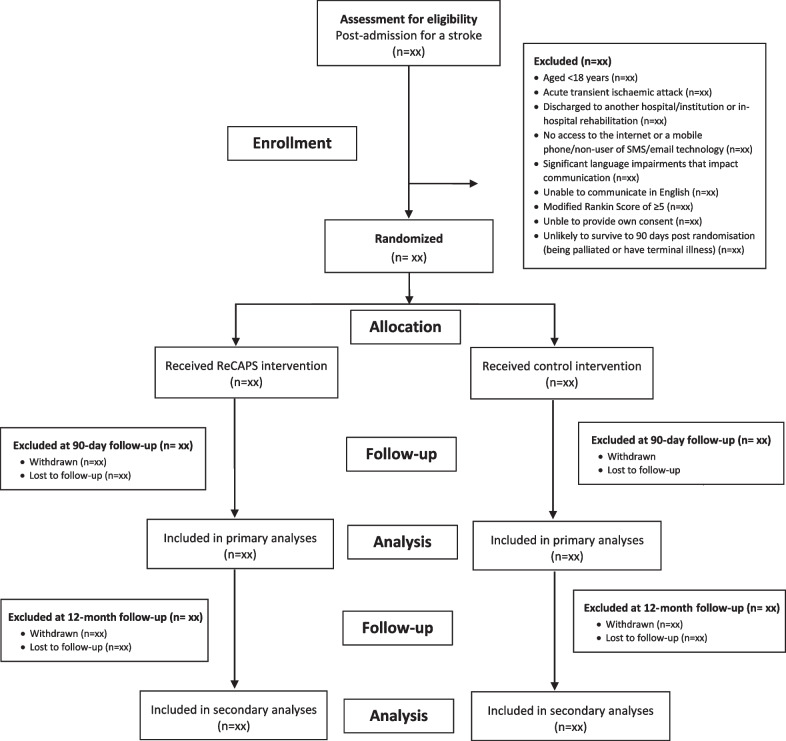
CONSORT flow diagram

To determine the representativeness of the trial cohort, we will compare characteristics of patients who were screened with those who participated. At the end of the trial, we will obtain a list of AuSCR registrants from participating hospitals that met the eligibility criteria to compare characteristics of those included in the trial with those eligible but who did not participate. Details of recruitment and eligibility criteria are described in the trial protocol [5], and information on eligibility, recruitment, and withdrawal/follow-up will be reported in a CONSORT flow diagram (Fig. 1). Variables to be requested will include demographic (e.g. sex, age) and clinical data (type of stroke, time since stroke, history of previous stroke). These data will be provided to the Monash staff using the project ID number allocated by AuSCR data custodians, excluding personal identifying variables.

### Baseline patient characteristics

Data on baseline characteristics will be summarised as frequencies and proportions for categorical variables and medians and interquartile ranges for continuous variables (Supplemental Table II).

## Analysis

### Outcome definitions

#### Primary outcome

The primary outcome is the proportion of participants who report having an “unplanned” hospital presentation (emergency department/admission) within 90 days following randomisation. This will be determined via self-report and confirmed through linkage with hospital data, as described below.

##### Self-report

Self-reported primary outcome data will be verified in the medical records obtained from the hospital where the participant was recruited. Emergency department presentations will be captured for any health condition, or a complication of stroke, and are assumed to be unplanned. Hospital admission will be coded as unplanned if they are not clearly defined or flagged as planned, or meet any of the clinical conditions as recommended by the Australian Institute of Health and Welfare [12] and outlined below:

- At risk of serious morbidity/mortality and requiring urgent assessment and/or resuscitation
- Have suspected organ failure or system failure
- Have an illness or injury where the viability of a body part organ is acutely threatened
- Have severe pain where the viability or function of an organ is suspected to be acutely threatened
- Have significant haemorrhage and requiring urgent assessment and treatment
- Have an acute condition which represents a significant threat to the patient’s physical or psychological wellbeing
- Have gynaecological or obstetric complications

After the trial is completed, an independent adjudication committee, who will remain unaware of the group allocation, will undertake a blinded adjudication of all hospital admissions within the 90-day post-randomisation period to ascertain whether admissions were planned or unplanned.

##### Administrative linked data

There will be linkage of trial participants with emergency department and hospital administrative data. Using these data, admission will be defined as “unplanned” if coded as “emergency admission — N1”, as recommended by the Australian Institute of Health and Welfare [12], or hospital admission within 24 h for any for the clinical conditions outlined above.

#### Secondary outcomes

The following secondary outcomes are being assessed at different points in the trial (Table 1):

1. Weighted goal attainment scale (GAS) T-score is calculated based on data obtained across five domains (i.e. participant’s health, mind and body, everyday activities, out and about, and healthcare and support) using the GAS questionnaire [13].
2. Change in the adoption of self-efficacy skills is being assessed using the Stroke Self-Efficacy Questionnaire. This 13-item questionnaire is used to collect information on the confidence of participants regarding undertaking tasks that may have been difficult due to the stroke [14].
3. Change in mood, i.e. anxiety and depression, is being measured using the Hospital Anxiety and Depression Scale [15].
4. Number of hospital contacts at 90 days post-randomisation: Composite outcome of number of self-reported emergency presentations or hospital admissions to be determined using self-reported or linked data.
5. Change in health-related quality of life is being assessed using the EQ-5D-3L questionnaire [16], across five health domains (i.e. mobility, self-care, usual activities, pain/discomfort, and anxiety/depression), and overall using a visual analogue scale.
6. Cost-effectiveness: Cost at 90-day and 12-month post-randomisation (self-reported resource use and/or administrative health service use data) per Quality Adjusted Life Year (QALY; derived from EQ-5D-3L questionnaire) gained.
7. Patient education and self-management skills attainment at 90 days post-randomisation is being assessed with the Health Education Impact Questionnaire [17].
8. Composite outcome at 90 days and 12 months post-randomisation: recurrent stroke, cardiovascular events or deaths. This will be determined using a combination of self-reported and linked data. Further details on analyses using linked data will be reported separately.
9. Resource utilisation/costs will be measured using data on resource use that were self-reported and/or obtained from linked administrative records. Details on analyses of this outcome will be reported separately.
10. Disability at 90-day post-randomisation assessed using the modified Rankin Scale [18].
11. Unmet needs at 90-day post-randomisation: assessed using the Longer-term Unmet Needs after Stroke (LUNS) questionnaire [19].

### Analysis methods

All analyses will be based on the intention to treat principle, where participants will be analysed according to the group in which they were allocated, regardless of whether or not they received the intervention or deviated from the protocol. The proposed format for presenting study outcomes is shown in Tables 2 and 3.

**Table 2:** Within- and between-group differences in primary outcome and categorical secondary outcomes

	Control (*N* =)			Intervention (*N* =)			OR (95% *CI*)^b^
	Baseline *n* (%)	90 days *n* (%)	Phi^a^	Baseline *n* (%)	90 days *n* (%)	Phi^a^	
Primary outcome							
Hospital presentation^c^	-		-	-		-	
Secondary outcomes							
Composite outcome^d^							
Problems in EQ-5D-3L dimension							
Mobility							
Self-care							
Usual activities							
Pain or discomfort							
Anxiety or depression							
Long-term unmet needs							

*OR* odds ratio, *CI* confidence interval, *EQ-5D-3L *EuroQol five dimensions three level. ^a^Measure of magnitude of within-group change at 90-day post-randomisation relative to the baseline measurement. ^b^Between-group difference at 90-day post-randomisation, adjusted for baseline measurements, clustering by recruitment hospital, and stratifying variables. ^c^Includes emergency department presentations and unplanned hospital admission. ^d^Comprises recurrent stroke, cardiovascular events, or deaths

**Table 3:** Within- and between-group differences in non-categorical secondary outcomes

	Control (*N* =)			Intervention (*N* =)			Difference (95% *CI*)^c^
	Baseline Mean (SD)^a^	90 days Mean (SD)^a^	Cohen’s D^b^	Baseline Mean (SD)^a^	90 days Mean (SD)^a^	Cohen’s D^b^	
*Self-efficacy score*							
*Goal attainment scaling (T score)*							
Your health							
Mind and body							
Everyday activities							
Out and about							
Healthcare and support							
*Health education impact*							
Positive and active engagement in life							
Health-directed behaviour							
Skill and technique acquisition							
Constructive attitudes/approaches							
Self-monitoring and insight							
Health service navigation							
Social integration and support							
Emotional wellbeing							
*Mood*							
HADS depression							
HADS anxiety							
*EQ-5D-3L VAS*							
*Number of hospital presentations*							
Emergency department presentations							
Hospital admissions							
Modified Rankin Scale							

*SD* standard deviation, *CI* confidence interval, *HADS* Hospital Anxiety and Depression Scale, *EQ-5D-3L VAS* EuroQol five dimensions three level visual analogue scale. ^a^Data may also be presented as median and interquartile range, depending on the distribution of the outcome. ^b^Measure of magnitude of within-group difference at 90-day post-randomisation relative to the baseline measurement. ^c^Between-group difference at 90-day post-randomisation, adjusted for baseline measurements, clustering by recruitment hospital, and stratifying variables

#### Primary outcome

The primary outcome will be compared between groups using a mixed-effects logistic regression model, adjusted for clustering by recruitment hospital (random effect) and stratification variables, i.e. age and the level of disability (modified Rankin Scale) at baseline. The primary outcome model will be adjusted for stratification variables through either direct adjustment or inverse probability of treatment weighting, depending on the final sample or convergence of the models [20, 21]. If there are convergence issues, we will undertake an inverse probability of treatment weighting, involving the use of covariate values to predict the probability of participants being allocated to their respective group. This approach will be used to create a weighted trial sample, in which study groups have a similar distribution of the covariate values. The weights will then be applied to a simple mixed-effects logistic regression model.

#### Secondary outcomes

Generalised mixed-effects regression models (including linear, logistic, quantile or negative binomial regression) will be used to compare secondary outcomes between allocation groups, depending on the nature and distribution of these outcomes. Models will be constructed using similar procedures specified for the primary outcome analysis. For comparison of changes in a secondary outcome measure from baseline between groups, regression models will comprise the outcome measure at the time of follow-up as the dependent variable, the group allocation as the independent variable, and the baseline measure of the outcome as a covariate. The magnitude of change from baseline within groups will be estimated as Cohen’s d for continuous or ordinal variables, or Phi co-efficient for categorical variables, based on outputs from the regression models.

### Sensitivity/subgroup analyses

We will undertake per-protocol analyses (described above) for all outcomes. Further, a limited number of unadjusted subgroup analyses will be undertaken regardless of the effect of the intervention on the primary outcome. These include analyses stratified by age (< 65 years *vs*. ≥ 65 years), sex (male *vs*. female), level of disability (modified Rankin Scale score ≤ 2 *vs*. > 2), living condition (living alone *vs*. with carer/family), educational attainment (university education *vs*. no university education), preferred mode of communication (SMS *vs*. email), and number of goals set (≤ 3 *vs*. > 3 goals). Apart from these pre-specified subgroup analyses, exploratory analyses may also be undertaken, informed by variables with significant statistical interaction with the intervention.

### Missing data

The primary analysis will be reported without imputation of missing data. However, if the proportion of missing primary outcome measure exceeds 10%, we will undertake multiple imputation of these missing data. This will involve multivariate imputation by chained equations algorithms, where the imputed value is conditional on observed values of other baseline variables [22]. This algorithm will be repeated for up to 20 cycles to obtained imputed values for the first imputed dataset. To ensure robustness of this imputation process, this process will be repeated to obtain 20 imputed datasets. The pooled estimate from these imputed datasets will be reported and compared with unimputed primary outcome model. We will also explore other imputation approaches for the primary outcome, including imputing missing values as either 0 or 1. Missing secondary outcomes will be imputed using multivariate imputation by chained equations algorithms.

### Additional analyses

Process and economic evaluations, including analyses of linked data, will be reported separately.

### Harms

#### Adverse events and serious adverse events

Safety will be defined by the number and frequency of reported adverse events and serious adverse events related to the intervention and will be reported using a format shown in Supplemental Table III. Such adverse events will include falls or accidents requiring medical attention or presentations to hospital. Moreover, deaths, disability/incapacity, or other life-threatening or important medical events related to the intervention will also be reported.

### Statistical software

All analyses will be undertaken using Stata/SE 17.0 (StataCorp 2021).

## Current status of the trial

As of 20th of December 2023, 462 participants have been recruited and randomised. Recruitment to the trial was affected significantly by the COVID-19 pandemic and thereafter. Due to funding constraints, recruitment will conclude in December 2023. Data lock is anticipated for the second quarter of 2024, when all 90-day follow-up assessments should have been completed. Further details on analyses of longer-term outcomes to be determined through data linkage, including resource utilisation/costs within 12 months and composite (cardiovascular or death) outcomes, will be reported separately.

### Supplementary Information

**Additional file1: **Supplemental tables: **Supplemental Table I**. Intervention fidelity. **Supplemental Table II**. Characteristics of participants at baseline. **Supplemental Table III**. Adverse events and serious adverse events related to the intervention

**Additional file 2.**

## Data Availability

The datasets used and/or analysed during the current study are available from the corresponding author on reasonable request.

## Abbreviations

EQ-5D-3L: The European Quality-of-Life questionnaire
GAS: Goal Attainment Scale
HADS: Hospital Anxiety and Depression Scale
heiQ: Health Education Impact Questionnaire
iVERVE: Inspiring Virtual Enabled Resources following Vascular Events
LUNS: Longer-term Unmet Needs Scale
mRS: Modified Rankin Scale
RCT: Randomised controlled trial
ReCAPS: Recovery-focused Community support to Avoid readmissions and improve Participation after Stroke
REDCap®: Research Electronic Data Capture
SMART criteria/goals: Specific, measurable, achievable, realistic and time-bound
SMART-GEM: SMART Goal Evaluation Method
SMS: Short message service
SSEQ: Stroke Self-Efficacy Questionnaire
