# Supplementary material for: Statistical analysis plan for the Recovery-focused Community support to Avoid readmissions and improve Participation after Stroke randomised controlled clinical trial
Source: Trials. 2024 Jan 23;25:78. doi: 10.1186/s13063-023-07864-2 (PMC10804563; doi:10.1186/s13063-023-07864-2)
Supplement: Supplementary file 1 — Additional file1: Supplemental tables: Supplemental Table I. Intervention fidelity. Supplemental Table II. Characteristics of participants at baseline. Supplemental Table III. Adverse events and serious adverse events related to the intervention [file 13063_2023_7864_MOESM1_ESM.docx]

**Supplemental Material**

**Supplemental Tables**

Supplemental Table I. Intervention fidelity

Supplemental Table II. Characteristics of participants at baseline

Supplemental Table III. Adverse events and serious adverse events related to the intervention

| **Supplemental Table I.** **Intervention Fidelity** | |
| --- | --- |
|  | **Operational element in the ReCAPS study** |
| **Study Design** | - Standardised intervention with clear protocol - Plan to include adherence to intervention in analysis (per-protocol analysis undertaken in addition to ITT analysis) - Training checklist to structure the observation of training sessions being delivered. - Documentation template |
| **Training RAs/Intervention Providers** | - Use of standardised training sessions - Quality assurance checks of process and content of training sessions - Role play of intervention telephone calls / goal setting interviews during training sessions, feedback given - Further training delivered as required across study (e.g. change of staff) |
| **Delivery of Intervention** | - All of the goals set and scored will be quality checked on the SMART GEM tool - Use of a structured documentation process for intervention delivery - Observation of randomly selected first intervention consults of all trained RAs (quality assurance visit) - Observation of ~10% of randomly selected goal-setting intervention consult (quality assurance visit). [Suggest quarterly review of random selection of calls]. - Use of quality assurance form to document and correct deviation from intended delivery of study intervention [use of a goal-setting call checklist?] - Use of a form to capture the adaptations made in the delivery of the ReCAPs intervention (allowing for identification, tracking and monitoring of adaptations to protocol) |
| **Receipt of Intervention** | - Receipt of all scheduled components (presented as the proportion of participants who received 100% of scheduled elements; as well as the mean level of completeness) - Participant self-efficacy in implementing newly recommended behaviours or strategies - Audit of dispatch logs from the electronic messaging gateway |
| **Enactment of Intervention Skills** | - Measuring enactment throughout the study (and not just at the endpoint). An outside observer will observe delivery of goal-setting using a checklist. - Adaptations that occur to any protocol processes throughout the study. |

| **Supplemental Table II. Characteristics of participants at baseline** | | |
| --- | --- | --- |
| **Baseline Characteristics** | **Control**  **N=**  **n (%)** | **Intervention**  **N=**  **n (%)** |
| **Demographics** |  |  |
| Median age (Interquartile range) |  |  |
| <65 years |  |  |
| 65+ years |  |  |
| Female |  |  |
| Born in Australia |  |  |
| Married/with partner |  |  |
| Retired |  |  |
| University educated |  |  |
| **Living situation** |  |  |
| Live independently (mRS 0-1) |  |  |
| Own home or unit |  |  |
| **Location of recruitment hospital** |  |  |
| New South Wales |  |  |
| Queensland |  |  |
| South Australia |  |  |
| Victoria |  |  |
| Western Australia |  |  |
| **Type of stroke** |  |  |
| Ischaemic |  |  |
| Haemorrhagic |  |  |
| Undetermined |  |  |
| **Preferred SMS communication** |  |  |
| **Use of health services** |  |  |
| Private health insurance |  |  |
| Hospital and extras cover |  |  |
| Use community services |  |  |
| Allied care services in last 4 weeks† |  |  |
| **Self-reported medical history** |  |  |
| Hypercholesterolaemia |  |  |
| Heart attack |  |  |
| Atrial fibrillation |  |  |
| Hypertension |  |  |
| Sleep apnoea |  |  |
| Respiratory problems |  |  |
| Diabetes |  |  |
| Arthritis |  |  |
| Depression |  |  |
| Anxiety |  |  |
| Cancer |  |  |
| Other illness |  |  |
| **Lifestyle characteristics** |  |  |
| Smoking status |  |  |
| Current smoker |  |  |
| Past smoker |  |  |
| Never smoker |  |  |
| Physically active |  |  |
| Risky alcohol consumption |  |  |
| Healthy eating |  |  |
| >5 servings of vegetables daily |  |  |
| >2 servings of fruit daily |  |  |
| mRS, modified Rankin Scale | | |

| **Supplemental Table III. Adverse events and serious adverse events related to the intervention** | | |
| --- | --- | --- |
|  | Control (N=)  n | Intervention (N=)  n |
| Adverse events |  |  |
| Serious Adverse events |  |  |
| Deaths |  |  |
